# Arizona Surge Line: An emergent statewide COVID-19 transfer service with equity as an outcome

**DOI:** 10.3389/fpubh.2022.1028353

**Published:** 2023-01-25

**Authors:** Lisa Villarroel, Erin Tams, Luke Smith, Jessica Rigler, Dena Wilson, Chengcheng Hu, Marilyn K. Glassberg

**Affiliations:** ^1^Division of Public Health Preparedness, Arizona Department of Health Services, Phoenix, AZ, United States; ^2^Phoenix Area Indian Health Service, Indian Health Service, Phoenix, AZ, United States; ^3^Epidemiology and Biostatistics, University of Arizona Mel and Enid Zuckerman College of Public Health, Tucson, AZ, United States; ^4^Medicine/Pulmonary, University of Arizona College of Medicine-Phoenix, Phoenix, AZ, United States; ^5^Pulmonary Medicine, Critical Care and Sleep Medicine, Banner-University Medical Center Phoenix, Phoenix, AZ, United States

**Keywords:** surge, COVID, load-leveling, transfer center, equity, access to care

## Abstract

**Introduction:**

The Arizona Surge Line was an emergent initiative during the COVID-19 pandemic to facilitate COVID-19 patient transfers and load-level hospitals on a statewide level. It was designed and implemented by the Arizona Department of Health Services in preparation for the first hospital surge due to COVID-19, recognizing the disproportionate impact that hospital surge would have on rural and tribal populations.

**Methods:**

We analyzed the Arizona Surge Line transfer data for the state's first two COVID-19 surges (4/16/2020–3/6/2021). Transfer data included transfer request characteristics, patient demographics and participating hospital characteristics. When applicable, we compared this data with Arizona census data, COVID-19 case data, and the CDC/ATSDR Social Vulnerability Index. The primary outcomes studied were the proportion of COVID-19 patient requests being successfully transferred, the median transfer time, and the proportion of vulnerable populations impacted.

**Results:**

During the period of study, 160 hospitals in Arizona made 6,732 requests for transfer of COVID-19 patients. The majority of these patients (84%, 95% CI: 83–85%) were placed successfully with a median transfer time of 59 min (inter-quartile range 33–116). Of all transfer requests, 58% originated from rural hospitals, 53% were for patients of American Indian/Alaska Native ethnicity, and 73% of patients originated from highly vulnerable areas. The majority (98%) of receiving facilities were in urban areas. The Arizona Surge Line matched the number of transfers with licensed market shares during the period of study.

**Conclusions:**

The Arizona Surge Line is an equity-enhancing initiative that disproportionately benefited vulnerable populations. This statewide transfer infrastructure could become a standard public health mechanism to manage hospital surges and enhance access to care during a health emergency.

## 1. Introduction

In the spring of 2020, public health officials across the country watched with concern as New York hospitals experienced the first COVID-19 surge in the United States. At that time, New York had no centralized infrastructure to coordinate and balance patient transfers between hospital systems. As a result, healthcare workers reported “apocalyptic” conditions in safety-net hospitals while private hospitals nearby had open beds. In the words of one observer, this was “a hospital system that was less than the sum of its mighty parts” (1, 2).

### 1.1. Background and rationale

Public health officials at the Arizona Department of Health Services (ADHS) feared emergency department crowding and imbalanced ICU patient flow in Arizona would similarly burden the hospital system and increase adverse outcomes, including patient mortality (3, 4). At that time, Arizona's system of hospitals, which includes private for-profit hospitals, public non-profit and public for-profit hospitals, critical access facilities, as well as the Indian Health Service and tribally operated P.L. 93–638 hospitals, had no existing centralized transfer infrastructure. Furthermore, Arizona health officials knew of no statewide precedent for such infrastructure.

There was a particular concern for the impact on rural and tribal populations in Arizona. Marginalized communities were already vulnerable to external health stressors and would soon face disproportionately higher rates of infection and mortality from COVID-19 (5). The bulk of the tertiary care system in Arizona lies within the urban centers and Phoenix and Tucson. If urban centers declined rural transfers due to overwhelmed capacity, a patient surge would likely worsen historical and ongoing health disparities in the state of Arizona.

### 1.2. Description of case

ADHS assembled the state's major hospital systems to implement a centralized statewide transfer infrastructure, the Arizona Surge Line (ASL), before the state's first COVID-19 surge. The ASL's mission was to protect hospitals and patients from unbalanced hospital surges by (a) load-leveling hospitals across the state, (b) expediting transfers of COVID-19 patients (c) ensuring equitable access to care, and (d) relieving clinicians of the operational burden of transfer to allow more time for patient care.

ADHS funded and administered the ASL. Hospital leaders contributed medical and transfer expertise to protocolize the ASL, focusing on bringing patients to a higher level of care (i.e., ICU). Patient transfers were directed to selected hospital systems based on a statewide bed availability dashboard and an alignment of transfer proportions with hospital systems' market share. Hospitals were not required to accept patients; final decisions on transfer and placement were made in real-time between physicians at the sending and receiving facilities. Large healthcare systems managed patient transfers within their system.

The ASL went live on April 16, 2020. In May 2020, the Arizona Governor issued an Executive Order that required hospitals to use the ASL for COVID-19 transfers and complete bed assignments within 30 min. It also required insurers regulated by the state to cover COVID-19 transfer and treatment at in-network rates if the ASL facilitated the transfer (6).

The rapid implementation and cost of the ASL are detailed in a prior publication (7). This manuscript details the outcomes from transfer requests for a higher level of care during the first two surges of COVID-19 in Arizona. When utilized in the ASL analysis, statewide census and COVID-19 data comes from the Arizona Census 2020 (8) and the ADHS COVID-19 dashboard, respectively (9). Data on social vulnerability comes from CDC/ATSDR's Social Vulnerability Index (SVI) (10).

## 2. Methods

### 2.1. Study design

The study is an evaluation of a public health initiative, and the Arizona Surge Line was not designed as a research project. This analysis could be classified as a retrospective cohort study.

### 2.2. Setting

Arizona is a large state (113,635 square miles) with distinct urban and rural regions. Within the state are a variety of private for-profit hospitals, public non-profit hospitals, public for-profit hospitals, Critical Access Hospitals, Indian Health Service and Tribally operated P.L. 93–638 Hospitals, and Veterans Affairs Medical Centers (VAMCs). Most hospital systems reside in Phoenix or Tucson. There is not a single hospital association that represents all hospitals.

The Arizona Department of Health Services (ADHS) is the state public health department, with a vision of “Health and Wellness for all Arizonans.” The Arizona Surge Line was a novel public health initiative for ADHS, as it centralized the transfer of COVID-19 patients and balanced patients across the state's varying hospital locations, systems, and types.

### 2.3. Study population

The study analyzed the data from all patient transfer requests through the Arizona Surge Line. This included patient and hospital demographics, along with the transfer outcomes.

### 2.4. Data variables and sources

Upon receiving a patient transfer request, the Arizona Surge Line captured minimal clinical information of the patient, which included patient demographics on transfer requests: patient name, date of birth, sex, home zip code, race/ethnicity, and COVID-19 status (positive or presumed positive); as well as the name of the sending facility and the level of care requested. ASL agents completed follow-up calls nightly between midnight to 4:00 a.m. to confirm the timing and outcome of patient placement.

### 2.5. Analysis

The analysis of the data was completed and validated by two independent investigators (E.T. and C.H.) to enhance validity and statistical rigor.

For the statistical analysis: count and percentage were used to summarize categorical variables, and median and inter-quartile range (IQR) used for continuous variables. Characteristics and outcomes were summarized for all transfer requests for the subgroup of completed transfers. Confidence interval (CI) for any proportion was obtained by the Clopper-Pearson method, and CI for any median by the exact method based on sign test. The unit of analysis was transfer request/patient. The score CI was used for difference of proportions.

## 3. Results

The data below reflect the following variables over Surges 1 (April 16, 2020 to September 19, 2020) and 2 (September 20, 2020 to March 6, 2021): (1) characteristics of the transfers requested and completed by the Arizona Surge Line, (2) demographics of the patients transferred through the Arizona Surge Line, and (3) characteristics of the Arizona hospitals transferring and receiving patients through the ASL.

### 3.1. Transfer characteristics

**Figure 1:**
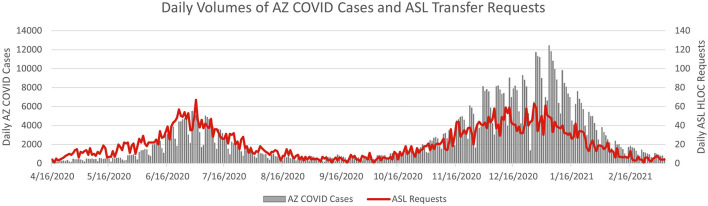
Daily counts of Arizona COVID-19 cases and ASL transfer requests to a higher level of care. In this graph, the daily volumes of ASL transfers (ASL data) are compared on top of the AZ COVID cases (state data) over the course of the first two COVID-19 surges in Arizona. Transfer requests generally followed the state's COVID-19 case counts, peaking at 67 requests/day surge Surge 1 and 63 requests/day during Surge 2.

During Surge 1 and Surge 2, hospitals across Arizona made 6,732 requests for transfer to a higher level of care. Hospitals without a parent system made up 73% of these requests. Transfer requests generally followed the state's COVID-19 case counts, peaking at 67 requests/day during Surge 1 and 63 requests/day during Surge 2 (Figure 1).

Most transfer requests were for ICU/ICU ECMO (40%; 95% CI: 39–41%) or Telemetry (36%; 95% CI: 35–38%) levels of care, with only 24% requesting Medical-Surgical placement (95% CI: 23–25%). Initial ASL protocols did not capture the requested level of care, yielding an 18% unknown result which was excluded from the proportion estimation for this characteristic. Known requested levels of care changed minimally between Surge 1 and Surge 2 with ICU/ICU ECMO requests increasing by 3% and Telemetry decreasing by 3.5% (Table 1).

**Table 1:** Characteristics and outcomes of Arizona Surge Line transfer requests: 4/16/2020–3/6/2021.

**All requested transfers**				
		**All**	**Surge 1**	**Surge 2**
**Number of transfer requests**		***N*** = **6,732**	***N*** = **2,880**	***N*** = **3,852**
Referring facility	Rural	3,904 (58%)	1,648 (57.2%)	2,256 (58.6%)
	Urban	2,676 (39.8%)	1,208 (41.9%)	1,468 (38.1%)
	Out of state	152 (2.3%)	24 (0.8%)	128 (3.3%)
Final case status	Canceled	942 (14%)	301 (10.5%)	641 (16.6%)
	Declined	154 (2.3%)	74 (2.6%)	80 (2.1%)
	Transferred	5,636 (83.7%)	2,505 (87%)	3,131 (81.3%)
Requested level of care^*^	ICU	2,114 (38.5%)	688 (37.1%)	1,426 (39.2%)
	ICU ECMO	87 (1.6%)	19 (1%)	68 (1.9%)
	Medical/surgical	1,298 (23.6%)	433 (23.4%)	865 (23.8%)
	Telemetry	1,991 (36.3%)	714 (38.5%)	1,277 (35.1%)
Patient race^#^	American Indian/Alaska Native	2,853 (49.5%)	1,353 (53.6%)	1,500 (46.3%)
	Asian	15 (0.3%)	8 (0.3%)	7 (0.2%)
	Black/African American	87 (1.5%)	54 (2.1%)	33 (1%)
	Hispanic	1,383 (24%)	628 (24.9%)	755 (23.3%)
	Native Hawaiian/Pacific Islander	9 (0.2%)	6 (0.2%)	3 (0.1%)
	White/Caucasian	1,420 (24.6%)	477 (18.9%)	943 (29.1%)
Patient gender^&^	Female	2,934 (43.7%)	1,295 (45%)	1,639 (42.6%)
	Male	3,787 (56.3%)	1,580 (55%)	2,207 (57.4%)
Patient age		61 (48, 72)	58 (44, 70)	63 (51, 73.8)
**All completed transfers**				
		**All**	**Surge 1**	**Surge 2**
**Number of completed transfers**		***N*** = **5,636**	***N*** = **2,505**	***N*** = **3,131**
Referring facility	Rural	3,486 (61.9%)	1,500 (59.9%)	1,986 (63.4%)
	Urban	2,056 (36.5%)	987 (39.4%)	1,069 (34.1%)
	Out of State	94 (1.7%)	18 (0.7%)	76 (2.4%)
Accepting facility	Rural	101 (1.8%)	33 (1.3%)	68 (2.2%)
	Urban	5,534 (98.2%)	2,471 (98.6%)	3,063 (97.8%)
	Out of state	1 (0%)	1 (0%)	0 (0%)
Time to place (minute)		59 (33, 116)	53 (29, 102.8)	64 (35, 126)
Requested level of care^*^	ICU	1,723 (37.1%)	565 (35.2%)	1,158 (38.1%)
	ICU ECMO	30 (0.6%)	12 (0.7%)	18 (0.6%)
	Medical/surgical	1,120 (24.1%)	373 (23.2%)	747 (24.6%)
	Telemetry	1,770 (38.1%)	657 (40.9%)	1,113 (36.7%)
Patient race^#^	American Indian/Alaska Native	2,578 (52.8%)	1,253 (56.3%)	1,325 (49.9%)
	Asian	11 (0.2%)	5 (0.2%)	6 (0.2%)
	Black/African American	65 (1.3%)	37 (1.7%)	28 (1.1%)
	Hispanic	1,131 (23.2%)	536 (24.1%)	595 (22.4%)
	Native Hawaiian/Pacific Islander	9 (0.2%)	6 (0.3%)	3 (0.1%)
	White/Caucasian	1,087 (22.3%)	388 (17.4%)	699 (26.3%)
Patient gender^&^	Female	2,501 (44.4%)	1,134 (45.3%)	1,367 (43.7%)
	Male	3,134 (55.6%)	1,370 (54.7%)	1,764 (56.3%)
Patient age		61 (47, 72)	58 (44, 70)	63 (51, 73)

The following table lists the characteristics and outcomes from the requested and completed patient transfers through the Arizona Surge Line from 4/16/2020 to 3/6/2021. This reflects the first two hospital surges due to COVID-19 in Arizona.

Surge 1: 4/16/2020–9/19/2020; Surge 2: 9/20/2020–3/6/2021.

Median (IQR) for continuous variables and count (percentage) for categorical variables.

* Requests without requested level of care captured (1,242 transfer requests including 993 completed transfers) were not included in the total count for proportion estimation; the initial ASL protocols did not capture this information.

# Requests without patient race (965 transfer requests including 755 completed transfers) were not included in the total count for proportion estimation.

& Requests without gender information (11 transfer requests including 1 completed transfers) were not included in the total count for proportion estimation.

The majority of requests resulted in successful placement in an acute care hospital. Accepted patient transfers made up 84% of request outcomes (95% CI: 83–85%) during Surge 1 and Surge 2. Cancellations represent 14% of request outcomes (95% CI: 13–15%) and occurred when placement was found within the referring system, at an out-of-state hospital, or was no longer required. There were 2% of requests declined due to available capacity or the availability of scarce resources such as ECMO and hemodialysis (Table 1).

The median of transfer time, defined as the interval between the first call to the ASL and ultimate bed assignment at a receiving facility, was 59 min (95% CI: 57–61) during the period of study; it increased from 53 min during Surge 1 (95% CI: 51–56) to 64 min in Surge 2 (95% CI: 61–67), as the capacity and resources of Arizona hospital systems became strained. At each surge peak, multiple facilities had to be contacted before the patient's placement was secured. During Surge 1 (excluding the initial week of the ASL program), the median transfer time over any 7-day period reached the highest level of 75.5 min during the 7-day period starting 7/3/2020, coinciding with the peak of COVID-19 cases in this surge; during Surge 2 the 7-day median reached the highest level of 118.5 min during the 7-day period starting 12/26/20, coinciding with the peak of Surge 2.

### 3.2. Patient demographics

The ASL captured the age of every transferred patient. During Surge 1 the median age for patients transferred was 58 years and comprised 55% male and 45% female. During Surge 2 the median age increased to 63 years with males comprising 56% of transfers and females 44%. These demographics align with data captured on Arizona's COVID-19 hospitalizations through Surge 1 and Surge 2 where hospitalizations by gender showed 53% male, 47% female, and an increasing percentage of patients 65 and older (Surge 1: 37%, Surge 2: 50%).

Race/ethnicity information for transferred patients was captured for 87% of completed transfers. Race/ethnicity is not a demographic that is typically collected during the hospital transfer process, but ASL data quality improved as sending facilities became more familiar with its process. After excluding the transfers which did not include race data, 53% were American Indian/Alaska Native (AI/AN), 23% Hispanic, 22% White/Caucasian, and 2% Black/African American, Asian, or Native Hawaiian/Pacific Islander. There was a 6-percentage point decrease (95% CI: 4–9%) in AI/AN representation from Surge 1 (56%) to Surge 2 (50%), with White/Caucasian increasing by 9% (95% CI: 7–11%) from Surge 1 (17%) to Surge 2 (26%).

**Figure 2:**
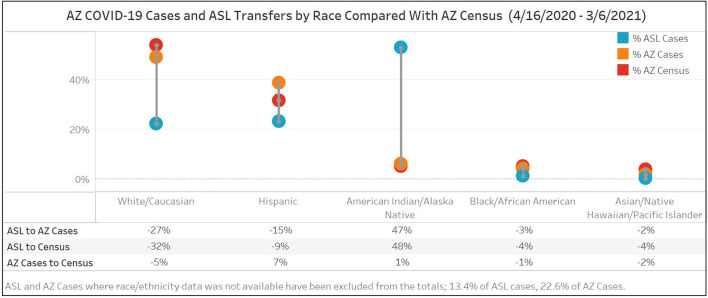
Race/ethnicity of Arizona COVID-19 cases and ASL completed transfers for Surge 1 and Surge 2 (4/16/20-3/6/21) compared to Arizona's 2020 Census. Race/ethnicity from Arizona's 2020 census is compared with Arizona's reported COVID-19 cases and ASL completed transfers during Surge 1 and Surge 2. The highest discrepancy between race proportions in ASL completed transfers and census was seen for AI/AN and White/Caucasian.

Race/ethnicity from Arizona's 2020 census is compared below (Figure 2) with Arizona's reported COVID-19 cases and ASL completed transfers during Surge 1 and Surge 2 (April 16, 2020 to March 6, 2021). The highest discrepancy between race proportions in ASL completed transfers and census was seen for AI/AN and White/Caucasian. AI/AN make up 5% of Arizona's population and 6% of AZ COVID cases yet disproportionately accounted for 53% (95% CI 51–54%) of the patients transferred through the ASL. An inverse relationship was seen for White/Caucasians who account for 55% of the state's population and 54% of AZ COVID-19 cases, but 22% of ASL transfers (95% CI 21–23%). Of note, 77% of Arizona COVID-19 case data included race/ethnicity data.

**Figure 3:**
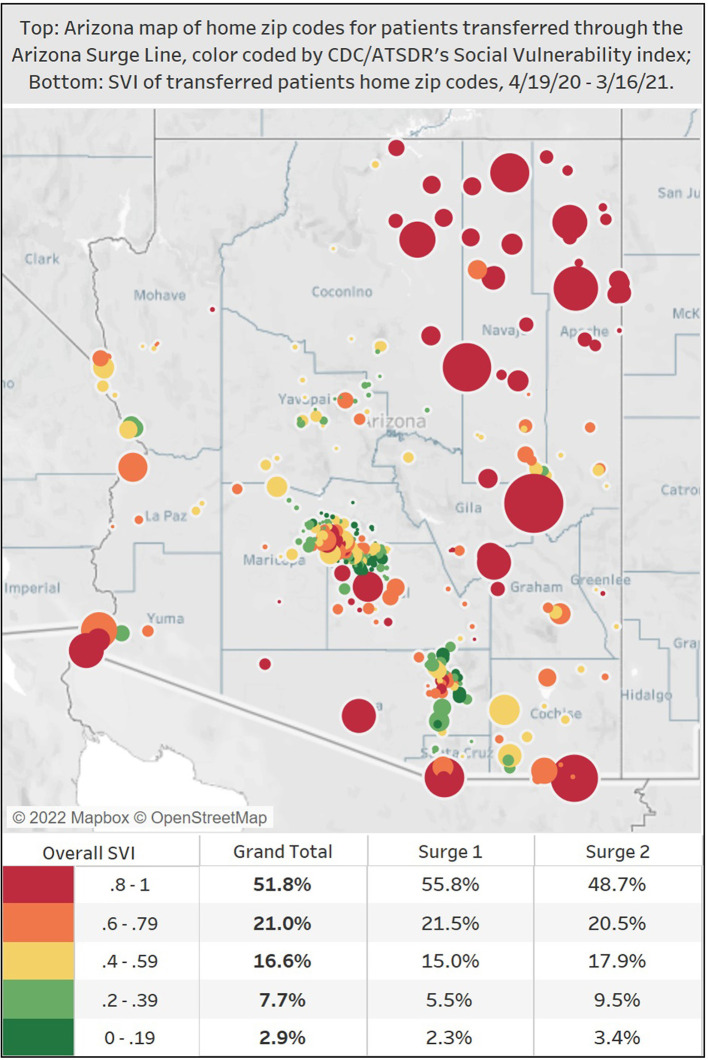
Map of home zip codes for transferred patients through the Arizona Surge Line (4/19/20–3/16/21), color-coded by CDC/ATSDR's Social Vulnerability Index. On this map of Arizona, the colors reflect the levels of the CDC/ATSDR social vulnerability index, with red and orange indicating the top two quintiles indicating greater vulnerability. The circles represent an ASL transferred patient's home zip code, and the larger the circle, the larger the transfer volume from that particular zip code. Zip codes with the highest vulnerabilities were the points of origin for 73% of ASL's transferred patients.

Patient home zip codes were collected from 92% of the ASL transfers with zip codes located outside the state of Arizona excluded from the analysis. Of these, 25% were P.O. Box zip codes, of which 79% were in reservation areas. Transfers that listed a P.O. Box zip code for patient origin treated the referring hospitals as a proxy for the home zip code. Zip codes with the highest vulnerabilities as measured on the CDC/ATSDR Social Vulnerability Index, which tracks factors that weaken a community's ability to prevent suffering and loss in a disaster, were the points of origin for 73% of the ASL patients' transfers (Figure 3).

Per the Executive Order, insurance coverage of COVID-19 patient transfer and care would be covered at in-network rates. The ASL did not collect information on insurance.

### 3.3. Hospital characteristics

In total, over 160 acute care hospitals within Arizona participated in the ASL, including 53 non-profits, 39 for-profits, 3 Veterans Affairs, 11 Indian Health Service, 10 tribally-operated facilities under Public Law 93–638 (P.L. 93–638), and 14 critical access hospitals. These spanned all 15 Arizona counties. An additional 31 clinics and post-acute care facilities also sought direct admission into Arizona hospitals during this time period.

**Figure 4:**
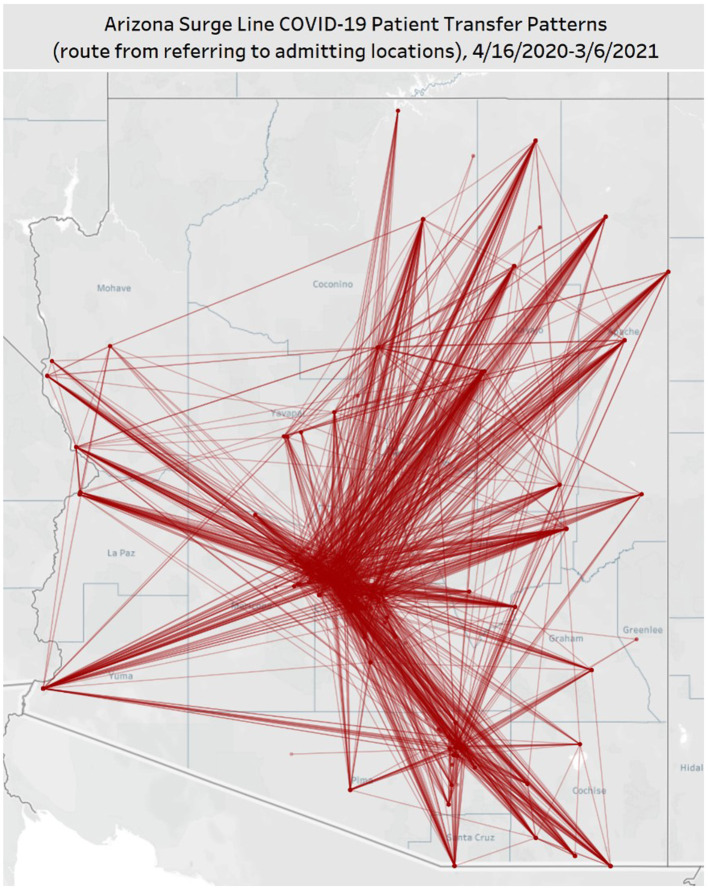
Map of hospital-to-hospital transfers completed through the ASL for Surge 1 (4/16/20–9/19/20) and Surge 2 (9/20/20–3/6/21). In this map of Arizona, each line represents a route patients took between sending and receiving facilities, as facilitated by the Arizona Surge Line. The time frame represents the first two COVID-19 surges in Arizona. 61% of transfers went from rural to urban facilities.

The majority (58%) of transfer requests were sent from Arizona's rural hospitals [classified the Health Resources & Services Administration definition (11)], with 30% coming from an Indian Health Service location, 31% from a critical access hospital, 29% from a tribally-operated P.L. 93–638, and 10% from a profit/non-profit/clinic. The majority (98%) of receiving facilities were in urban areas. Figure 4 shows the movement of patients during Surge 1 and 2, with 61% of transfers going from rural to urban facilities. Only 2% of transfers were rural to rural, an abnormal transfer pattern that occurred during peak surges when urban facilities had no more available resources.

Transfer requests from out-of-state hospitals comprised a small percentage of the total ASL requests. The ASL did not facilitate these transfers when Arizona hospital capacity was < 10%. Out-of-state transfer requests increased from Surge 1 (0.8%) to Surge 2 (3.3%).

The algorithms for patient placement for the ASL included consideration for hospital system licensed market share. During Surge 1 and Surge 2, the Arizona Surge Line successfully aligned the percentage of transferred patients to within 5% of the licensed market share for all systems.

Throughout Surges 1 and 2, no Arizona hospitals reported through state-required reporting to be functioning under crisis standards of care (internal ADHS data).

## 4. Discussion

### 4.1. Summary of findings

During Surges 1 and 2, the ASL wove a patchwork of private and public hospital systems with varying capacities and resources into a support network that exceeded the sum of its parts. To our knowledge, the ASL is the only statewide load-leveling transfer line in the country that has documented this level of systematic equity enhancement, patient placement, and hospital load leveling.

During the period of study, rural facilities initiated the majority of transfer requests. The predominance of rural requests bears witness to the uneven distribution of healthcare infrastructure such as ICU/ICU ECMO and telemetry beds in the state and specialty services such as pulmonology or nephrology. The increase in out-of-state transfer requests is likely due to the increased lack of hospital capacity in the surrounding states, and suggests a regional approach to load-leveling may be indicated.

The patients transferred were predominantly American Indian/Alaskan Native (AI/AN), with a downward trend between Surges 1 and 2 likely attributable to the resourceful approaches to vaccination campaigns conducted in and by those populations in Arizona (12). The disproportionate transfers from AI/AN communities and socially vulnerable zip codes speak to the movement of patients who lack a larger parent healthcare system.

Except in the direst circumstances, urban facilities received these patients. Although transfers never consistently met the target of 30-min bed placement, most requests were fulfilled, and ASL transfers were anecdotally reported to be faster than non-COVID-19 placement. The ASL transfers successfully matched the market share benchmarks for participating organizations, relieving concerns of some hospital systems that transfer through the ASL may negatively impact market value.

### 4.2. Prior research

During the pandemic, a growing body of studies, guidelines, and recommendations on hospital surge management have emphasized load-balancing to enhance equity, improve patient outcomes, and postpone or mitigate crisis standards of care (13–16). Three regional transfer lines, including Arizona's, jointly recommended the implementation of regional, centralized transfer centers as a fundamental component of a surge response (17). To our knowledge, this is the first published set of outcomes from a statewide load-leveling and transfer center in this country.

### 4.3. Lessons learned

The ASL, a statewide load-leveling and transfer center, is an equity-enhancing initiative. It removed insurance as a barrier, expedited transfers for patients from vulnerable zip codes and from populations disproportionately impacted by COVID-19, and supported rural facilities and providers. Further, while no causation can be inferred, there was no widespread hospital activation of crisis standards of care or triage during the period of study. We anticipate critical medical review of this initiative will show that the Arizona Surge Line saved lives, especially among the marginalized populations with disparities in excess mortality associated with COVID-19.

Based on the successful outcomes in Arizona during Surges 1 and 2, transfer infrastructure should be a standard public health mechanism to anticipate and diffuse potential hospital surges in future health crises.

### 4.4. Limitations/acknowledgment of methodological or conceptual constraints

This analysis was limited to the patients that were transferred between Arizona hospital systems through the Arizona Surge Line, and thus does not include out-of-state transfers, non-COVID transfers or COVID transfers that occurred within a hospital system.

Additionally, this analysis was limited to the first two COVID-19 hospital surges in Arizona. The Arizona Surge Line functioned differently in the third surge and should be addressed separately.

## Data availability statement

The original contributions presented in the study are included in the article/supplementary material, further inquiries can be directed to the corresponding author.

## Ethics statement

Ethical review and approval was not required for the study on human participants in accordance with the local legislation and institutional requirements. Written informed consent from the participants' legal guardian/next of kin was not required to participate in this study in accordance with the national legislation and the institutional requirements.

## Author contributions

LV wrote the first draft. ET and CH completed the analysis. LS, DW, JR, and MG wrote and adjusted sections of the manuscript. All authors contributed to manuscript revision, read, and approved the submitted version.
